# Secondary Fahr Syndrome Presenting With In-Flight Generalized Status Epilepticus in a Middle-Aged Male

**DOI:** 10.7759/cureus.83114

**Published:** 2025-04-28

**Authors:** Ali Almarzooqi, Samar Iltaf Mairajuddin, Javeed A Ahmad Dar, Abubaker Abdul Rahman Shaffi Al Madani

**Affiliations:** 1 Neurology, Rashid Hospital, Dubai, ARE

**Keywords:** fahr’s disease or fahr’s syndrome, fahr's syndrome, marijuana use, secondary fahr's syndrome, status epilepticus (se)

## Abstract

Fahr syndrome is a rare neurological condition characterized by bilateral basal ganglia calcifications, typically associated with metabolic disturbances such as calcium-phosphorus imbalance or parathyroid dysfunction. Although often incidental or subclinical, it may present with a range of neuropsychiatric or seizure-related symptoms. Status epilepticus as an initial manifestation is uncommon and rarely reported. We report the case of a 42-year-old male with no prior seizure history who developed generalized status epilepticus during a long-haul international flight. Initial brain imaging revealed extensive bilateral basal ganglia calcifications. Laboratory investigations showed mild ionized hypocalcemia (0.99 mmol/L), secondary hyperparathyroidism, and vitamin D deficiency, with normal renal function. The patient admitted to smoking a significant amount of cannabis (13 joints) before boarding, which may have acted as a seizure trigger in the context of underlying metabolic vulnerability. Secondary Fahr syndrome was diagnosed based on clinical, radiological, and biochemical findings. This case highlights a rare but serious presentation of Fahr syndrome with in-flight generalized status epilepticus.

## Introduction

Fahr syndrome (FS), idiopathic basal ganglia calcification, is a rare neurological condition characterized by bilateral calcifications in the basal ganglia and other brain regions, such as the cerebellar dentate nuclei and thalamus [1]. It can be classified into primary (familial/idiopathic) or secondary forms. Secondary FS is more common and is typically associated with metabolic and endocrine disturbances such as hypoparathyroidism, hyperparathyroidism, or vitamin D deficiency [2].

The clinical presentation of FS is heterogeneous. It may be asymptomatic or manifest with movement disorders (e.g., parkinsonism, dystonia), cognitive impairment, neuropsychiatric symptoms, or seizures [3]. While epileptic seizures are reported in 30-40% of symptomatic cases, generalized status epilepticus as the initial presentation is extremely rare [4].

Here, we report a unique case of secondary FS in a previously healthy 42-year-old male who developed in-flight generalized status epilepticus. The episode was later linked to underlying mild hypocalcemia, secondary hyperparathyroidism, vitamin D deficiency, and possible triggering by heavy cannabis use. This case underscores the need for a high index of suspicion for metabolic and structural causes of seizures, particularly in atypical scenarios and younger individuals with no prior neurological history.

## Case presentation

A 42-year-old male with a background of type 2 diabetes mellitus and dyslipidemia presented with first-onset generalized tonic-clonic seizures during a 16-hour international flight from Los Angeles to Dubai. According to his wife, he had poor sleep duration (less than 5 hours) the night prior to his travel and became increasingly anxious during the final hours of the flight. Around 9:00 PM, she observed a staring spell and unresponsiveness, followed by a generalized seizure lasting approximately 10 minutes. The semiology was described as full body tonicity interrupted by clonic jerks, associated with up-rolling of the eyes, mouth frothing, and urinary incontinence, without tongue biting.

The patient experienced two additional seizures in the ambulance, each lasting 2-3 minutes. Between seizures, he was drowsy but could minimally respond to simple questions. In the emergency department, he was administered intravenous diazepam 5 mg and a loading dose of levetiracetam 1.5 g. His neurological examination revealed a Glasgow Coma Scale (GCS) of approximately 10 (E3V2M5), consistent with a postictal state, without focal motor deficits. Elective intubation was performed due to recurrent seizures, and a diagnosis of convulsive status epilepticus was made, based on International League Against Epilepsy (ILAE) criteria.

Upon stabilization and extubation in the intensive care unit (ICU), the patient was conscious, oriented, and neurologically intact aside from mild gait ataxia and slight limb incoordination. The ataxia was attributed to postictal cerebellar dysfunction and underlying metabolic disturbance, with an expected favorable prognosis.

Following extubation, the patient provided a reliable history. He denied alcohol use over the past decade and had no history of trauma. He reported habitual weekly marijuana use, estimated at approximately 1-2 g per week. On the day of travel, however, he consumed an estimated 3-5 g of cannabis across multiple sessions - a marked increase compared to his baseline usage. Heavy tetrahydrocannabinol (THC) ingestion can lower the seizure threshold, particularly in the setting of metabolic vulnerability, and was considered a contributing factor in this case.

Key metabolic laboratory results are summarized in Table 1, alongside reference ranges for clinical interpretation. Both ionized and serum calcium levels were measured multiple times and remained persistently below the normal range. Ionized calcium represents the biologically active fraction, whereas serum calcium includes protein-bound components. The combination of chronic mild hypocalcemia, secondary hyperparathyroidism, and elevated phosphate levels supported the diagnosis of secondary FS.

**Table 1:** Summary of Laboratory Investigations and Reference Ranges

Parameter	Result	Reference Range
Ionized calcium	0.99 mmol/L	1.15–1.29 mmol/L
Serum calcium	8.0 mg/dL	8.6–10.2 mg/dL
Parathyroid hormone (PTH)	69.7 pg/mL	15–65 pg/mL
Phosphate	4.6 mg/dL	2.5–4.5 mg/dL
Magnesium	2.76 mg/dL	1.5–2.5 mg/dL
Vitamin D	23 ng/mL	>30 ng/mL (sufficient)
Renal and liver function tests	Normal	–
Glucose and other electrolytes	Normal	–

**Figure 1:**
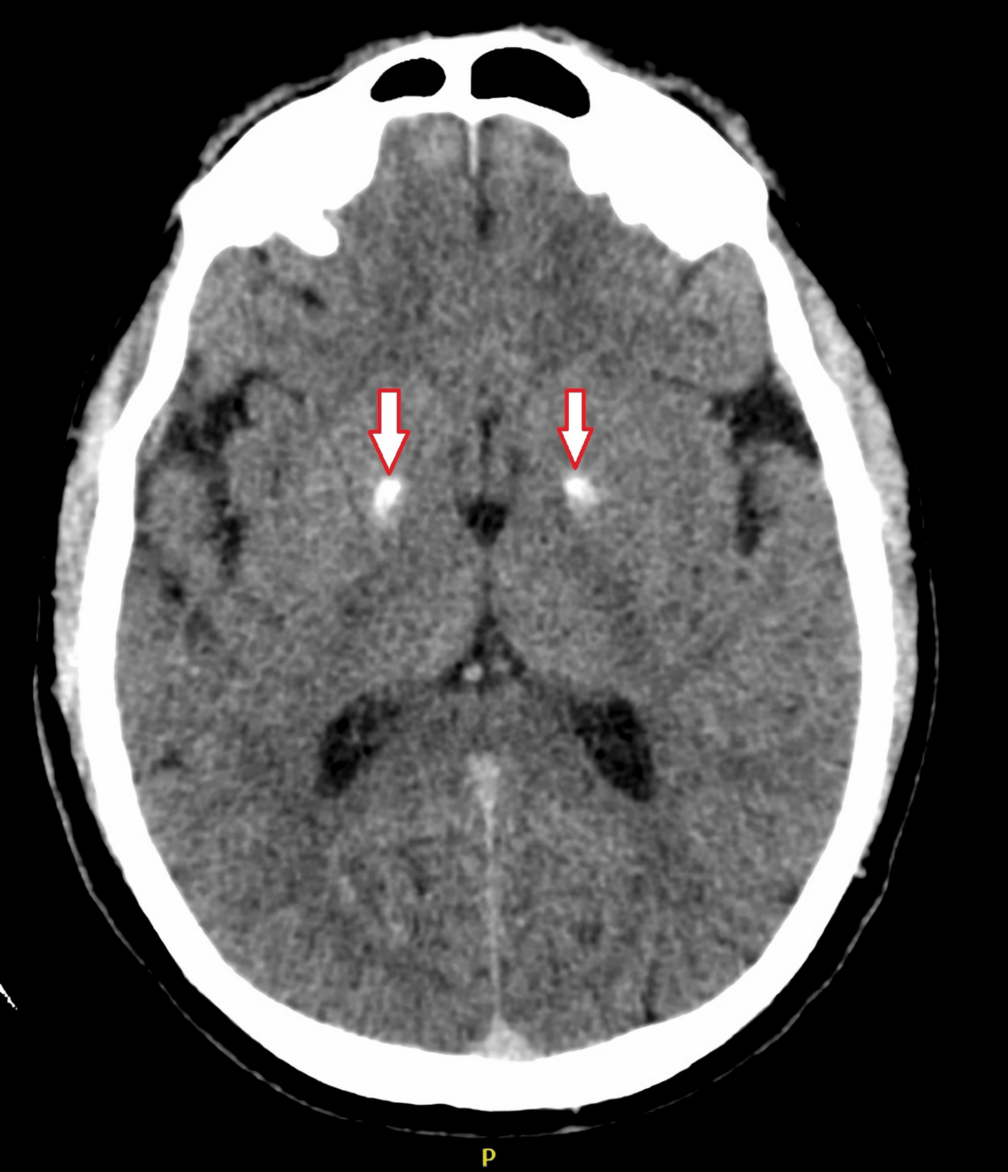
Non-contrast axial computed tomography (CT) of the brain showing bilateral symmetrical hyperdensities in the globus pallidus (arrows), consistent with calcium deposition.

**Figure 2:**
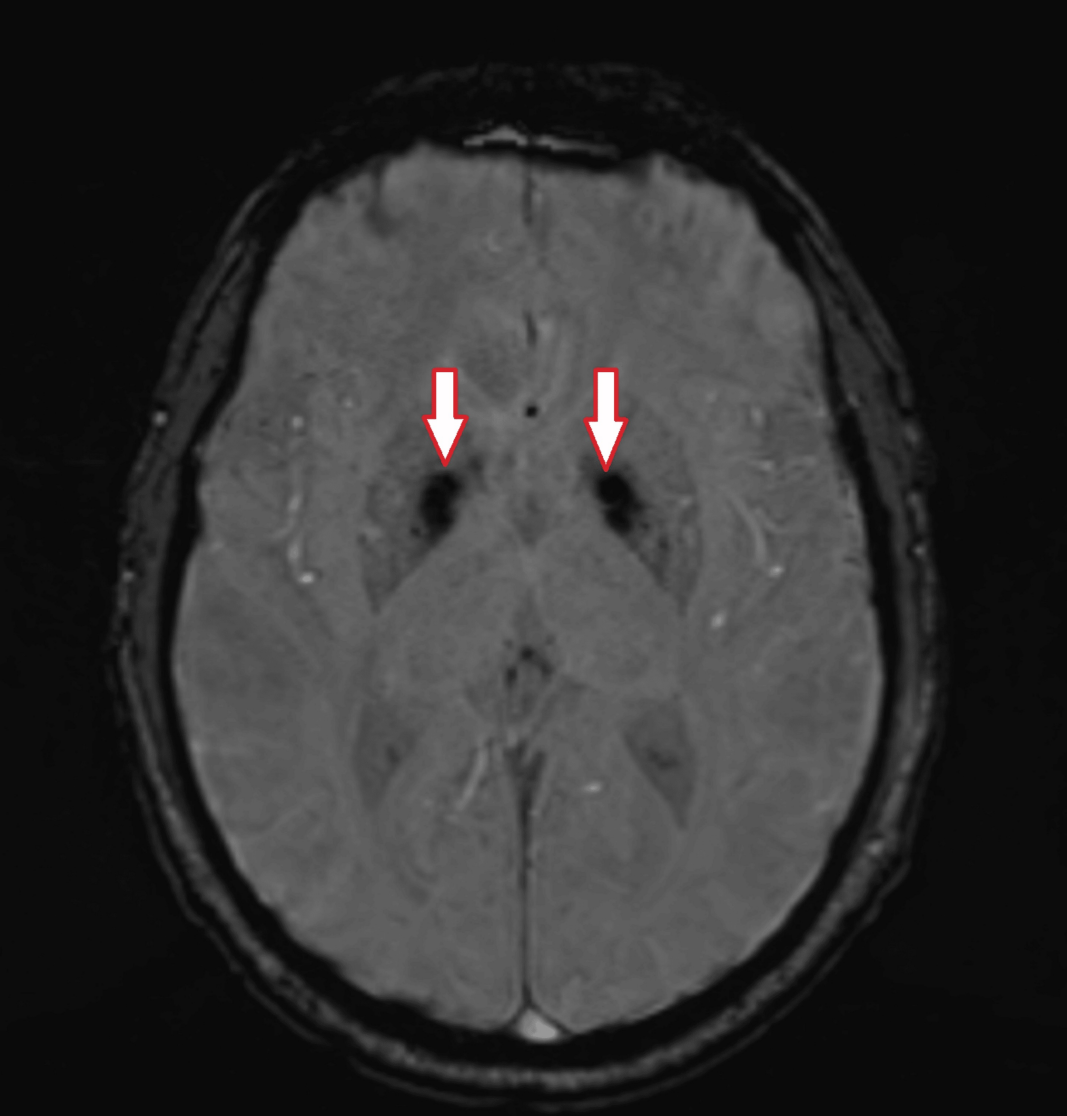
Axial susceptibility-weighted imaging (SWI) MRI of the brain demonstrating marked hypointensities in the bilateral basal ganglia, consistent with mineral (calcium) deposition (arrows).

Neuroimaging revealed extensive bilateral basal ganglia calcifications. Non-contrast computed tomography (CT) brain imaging showed bilateral symmetrical hyperdensities in the globus pallidus (Figure 1), and brain MRI with susceptibility-weighted imaging (SWI) demonstrated corresponding hypointensities consistent with mineral (calcium) deposition (Figure 2). A CT venogram ruled out cerebral venous thrombosis. Electroencephalogram done the next day demonstrated mild to moderate generalized nonspecific cerebral dysfunction without epileptiform discharges. Cerebrospinal fluid analysis and blood cultures were unremarkable.

The patient was diagnosed with secondary FS, likely associated with chronic mild hypocalcemia and secondary hyperparathyroidism. His seizures were interpreted as provoked, triggered by metabolic vulnerability, heavy cannabis intake, and sleep deprivation.

He was maintained on levetiracetam 500 mg twice daily and counseled regarding seizure precautions and lifestyle modifications. He was discharged with neurology and endocrinology follow-up. Upon discharge, he was fully oriented, ambulant, and seizure-free. The mild ataxia observed was attributed to postictal cerebellar dysfunction and underlying metabolic disturbances, and it showed gradual improvement, with an excellent prognosis for full recovery following metabolic correction and supportive therapy.

## Discussion

FS is a rare neurological condition characterized by bilateral basal ganglia calcifications and a broad spectrum of clinical manifestations. Symptoms may include pyramidal and extrapyramidal features, cognitive and behavioral disturbances, tremors, dystonia, and seizures. Although more commonly reported in young to middle-aged adults, FS can present at any age, and its occurrence in older adults has been described. This report illustrates the complex relationship between metabolic abnormalities and neurological symptoms, specifically in the context of secondary FS. Long-standing metabolic disturbances, such as hypocalcemia, secondary hyperparathyroidism, and vitamin D deficiency, may contribute to increased neurological vulnerability, which, when compounded by additional stressors, can precipitate severe presentations such as generalized status epilepticus. In this case, the imaging findings of bilateral basal ganglia calcifications, alongside biochemical abnormalities, supported the diagnosis of secondary FS, although multiple provoking factors, including heavy cannabis use and sleep deprivation, likely contributed to seizure onset.

Seizures are increasingly recognized as a neurological manifestation of FS, particularly when bilateral basal ganglia calcifications are present. While cognitive decline and movement disorders are the more classical manifestations, seizures - especially new-onset seizures - should prompt consideration of FS even in middle-aged individuals, such as this patient in his early 40s [5]. Generalized status epilepticus as a presenting feature of secondary FS remains exceptionally rare, but case reports highlight that metabolic disturbances such as hypocalcemia, often seen in conditions like pseudohypoparathyroidism [6], may lower the seizure threshold in susceptible individuals. Immediate seizure triggers in this patient included heavy cannabis consumption, significant sleep deprivation, and situational stress associated with long-distance travel [7], superimposed on his background of chronic metabolic vulnerability.

When encountering unexplained new-onset seizures in middle-aged patients, especially when imaging reveals basal ganglia calcifications, clinicians should consider secondary FS in the differential diagnosis [8]. A thorough metabolic workup - including serum calcium, phosphate, parathyroid hormone, and vitamin D levels - is critical for identifying treatable underlying causes.

From a therapeutic standpoint, this case emphasizes that targeted correction of metabolic abnormalities may help mitigate neurological symptoms and potentially delay the progression of calcifications. Early supplementation with calcium and vitamin D may stabilize biochemical parameters and improve neurological outcomes. Additionally, lifestyle factors-particularly cannabis use-can act as precipitants for seizures in metabolically vulnerable individuals, warranting careful counseling and monitoring [7].

Management primarily involved the neurology service during hospitalization. The patient was advised to follow up with endocrinology after discharge for further evaluation and management of his underlying metabolic disturbances. In broader presentations of FS, other specialties such as psychiatry, nephrology, or rehabilitation medicine may also be involved if clinically indicated [9].

Finally, although this case highlights important clinical lessons, limitations include the single-patient case report and lack of long-term follow-up. Further research is necessary to better characterize seizure risk in secondary FS and the potential modulating effect of metabolic correction.

## Conclusions

This case underscores the importance of considering secondary FS in the differential diagnosis of new-onset seizures, particularly in middle-aged patients with basal ganglia calcifications on neuroimaging. Metabolic disturbances such as hypocalcemia and secondary hyperparathyroidism should be promptly identified and corrected. Additionally, lifestyle factors such as cannabis use may further exacerbate seizure risk in metabolically vulnerable individuals. Early metabolic intervention and lifestyle counseling may improve neurological outcomes in secondary FS.
